# Major disturbances test resilience at a long‐term boreal forest monitoring site

**DOI:** 10.1002/ece3.5061

**Published:** 2019-03-15

**Authors:** James Weldon, Ulf Grandin

**Affiliations:** ^1^ SLU, Institutionen för vatten och miljö Uppsala Sweden

**Keywords:** bark beetle, boreal, disturbance, forest, ground vegetation, Norway spruce, resilience, storm

## Abstract

1. The impact of disturbances on boreal forest plant communities is not fully understood, particularly when different disturbances are combined, and regime shifts to alternate stable states are possible after disturbance. A long‐term monitored semi‐natural forest site subject to intense combined storm and bark beetle damage beginning in 2005 provided an opportunity to investigate the postdisturbance development of the vegetation community. Previous studies suggest that a shift from *Picea* *abies* to *Fagus sylvatica* domination was possible.
2. We analyzed pre‐ and postdisturbance vegetation data to investigate to what extent vascular plant species abundances, diversity, traits, and community composition have changed. We were particularly interested in differences between remaining apparently unaffected areas (potential refugia) and disturbed areas, and in signs of consistent change over time in community composition in response to disturbance that could indicate an impending regime shift.
3. We found that the vegetation community present in the refuge areas has remained substantially intact through the period of disturbance. Nonrefuge areas diverged from the refuges in community composition and showed increased taxonomic and functional diversity. Despite this, and an increase in deciduous tree species (particularly *F. sylvatica*), *P. abies* has shown strong postdisturbance regeneration. The refuges may be important in the apparent ongoing recovery of the disturbed areas to a *P. abies‐*dominated state similar to that found predisturbance. This fast recovery is interpreted as evidence of a system resilient to a potential shift to a deciduous‐dominated state.
4. *Synthesis*: Our results show that even powerful combined disturbances in a system with multiple stable states can be insufficient to initiate a regime shift. Resilience of the *P. abies*‐dominated forest community is increased by the survival of refuge areas functioning as a form of ecological memory of the previous ecosystem state. The results also demonstrate the value of data generated by long‐term monitoring programs.

## 1 INTRODUCTION

Forests free of human influence are almost entirely absent in Scandinavia, and unmanaged semi‐natural forest is rare, with most being managed for production (Östlund, Zackrisson, & Axelsson, 1997). While all forests are subject to disturbances, studying their impact in managed forest is complicated by the confounding effects of management regime (Hedwall & Brunet, 2016). The small remaining area of unmanaged semi‐natural forest in the region (i.e., forest composed predominantly of native species which have not been planted but which is not free of human influence) therefore presents an opportunity to study the effects of disturbances on natural processes of regeneration and succession. There is also a scarcity of scientific studies of the effects of disturbances in boreal forests where long‐term vegetation monitoring data are available. Monitoring programs are few, and disturbances unevenly distributed both temporally and spatially (Diaz‐Yanez, Mola‐Yudego, Eriksen, & González‐Olabarria, 2016).

In January 2005, a storm caused extensive damage to forests in southern Sweden, including the Aneboda monitoring site (part of the International Cooperative Programme on Integrated Monitoring of Air Pollution Effects on Ecosystems, under the UN Convention on Long‐Range Transboundary Air Pollution (ICP IM, 2018)). Around 20% of the Norway spruce (*Picea abies*)‐dominated forest was felled, followed by an outbreak of bark beetle (*Ips typographus*) which killed most of the remaining large spruce (Löfgren, Grandin, & Stendera, 2014). Despite this damage, the monitoring program continued, providing a unique opportunity to investigate the postdisturbance development of vegetation communities in semi‐natural forest.

Forest plant species have evolved subject to disturbances such as fire, wind, and insect outbreaks and have to some extent adapted to them (Gutschick & BassiriRad, 2003; Keeley, Pausas, Rundel, Bond, & Bradstock, 2011), which can increase resilience, for example, serotiny in fire‐prone ecosystems (Buma, Brown, Donato, Fontaine, & Johnstone, 2013). Indeed, disturbance has a fundamental role in shaping the development, structure, and function of forest ecosystems (Angelstam & Kuuluvainen, 2004), opening gaps, and initiating succession processes (Thom et al., 2017). Even after a stand‐replacing combination of storm damage and an outbreak of bark beetle (*Ips typographus*) which destroy the bark of mature spruce and introduce disease, some mature trees survive, providing a seed source (Kupferschmid & Bugmann, 2005) and facilitating the eventual regeneration of a similar canopy to that found predisturbance (Nováková & Edwards‐Jonášová, 2015). In addition, many understory plant species have been shown to persist as established plants, seeds, or rootstocks through wildfire, wind, and insect disturbances (Swanson et al., 2011).

However, forests also have the potential to develop along alternative successional pathways after perturbations (Taylor & Chen, 2011). Increased disturbance intensity can shift the expected regeneration pathway of a coniferous forest toward a deciduous‐dominated or grassland state for example (Johnstone, Hollingsworth, Chapin, & Mack, 2010). Combined disturbances can create alternative successional pathways. A North American pine forest regenerated as pine dominated after fire, as spruce/fir dominated after windthrow but birch dominated after windthrow followed by fire (Johnstone et al., 2016). The effects of such compound disturbances remain poorly understood (Trumbore, Brando, & Hartmann, 2015; Turner, 2010). In addition to disturbances such as storms and insect outbreaks, forests are also subject to more diffuse anthropogenic stress (Seidl et al., 2017). Nitrogen deposition originating in the combustion of fossil fuel and agricultural emissions (Bobbink et al., 2010) is a widespread problem (Jonard et al., 2015; Waldner et al., 2014) with the potential to change understory vegetation via eutrophication (Dirnböck et al., 2014; Hedwall & Brunet, 2016). In addition, many European spruce forests face increasingly unfavorable conditions due to changing climate (Falk & Hempelmann, 2013). Modeling of tree species distributions under climate change scenarios suggests that southern Sweden will be more suitable for deciduous broadleaved species than for spruce by the end of the century (Hanewinkel, Cullmann, Schelhaas, Nabuurs, & Zimmermann, 2013). As a result, disturbance‐induced shifts to beech (*Fagus sylvatica*) dominance may already be underway (Bolte, Hilbrig, Grundmann, & Roloff, 2014).

**Figure 1:**
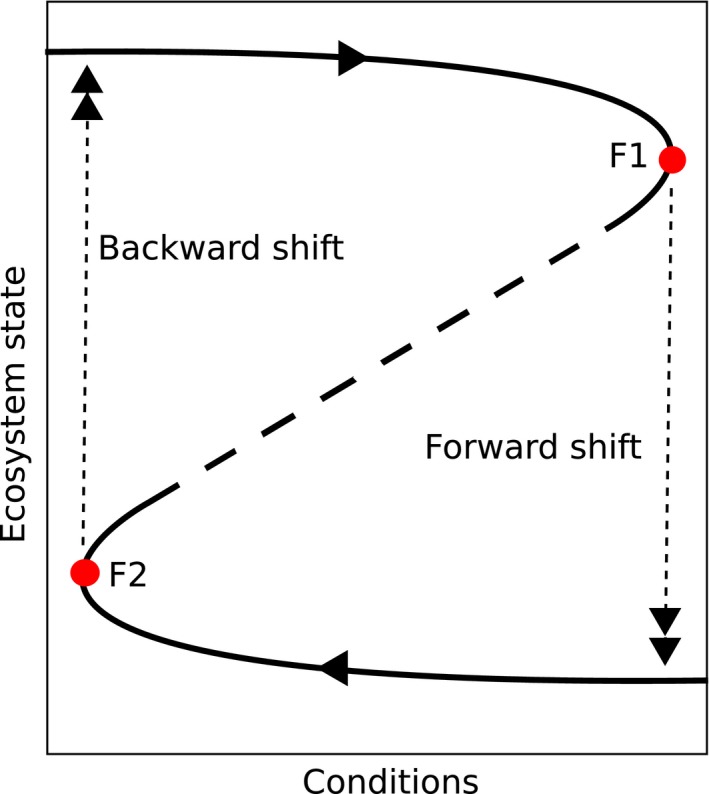
A system may reach a critical point (F2) via incremental changes, at which stage it forward shifts to a new stable state. However, to go backward, it is not enough to return to F2. Instead, the other inflection point at F1 must be reached. This inability to reverse along the same path is known as hysteresis (redrawn from Scheffer et al. (2001))

The theoretical basis for such regime shifts has been developed through the study of resilience and of ecological responses to disturbance (Holling, 1973). Various definitions of these terms have been made: Here, we follow those developed in a recent paper (Angeler & Allen, 2016) attempting to bring some clarity to this area. Ecological resilience can be simply defined as “a measure of the amount of change needed to change an ecosystem from one set of processes and structures to a different set of processes and structures” (Angeler & Allen, 2016). This change can be thought of as moving from one stable state (or basin of attraction) to another (Folke et al., 2004; Scheffer, Carpenter, Foley, Folke, & Walker, 2001). Once this shift has occurred, the end of the disturbance that caused the change is not enough to return the system to its predisturbance state (Holling, 1973). The same reinforcing processes that underlay the resilience to change of the system in its predisturbance equilibrium state then contribute to maintaining the system in its new, alternative equilibrium (Figure 1, Scheffer et al., 2001). In the context of this study, a regime shift could be a change from a spruce‐dominated forest to one dominated by broadleaved species.

Succession in boreal forest is a slow process, and the time needed for a return to a mature forest characterized by the dynamics of ongoing small gap formation and subsequent local succession processes can be over 300 years after a major disturbance (Kuuluvainen & Ankala, 2011). However, while the establishment of a late‐successional canopy takes many decades, changes in the ground vegetation can be observed on shorter timescales, and the regeneration of tree species there can to some extent suggests the composition of the future canopy (Heurich, 2009; Macek et al., 2016; Thrippleton, Bugmann, Kramer‐Priewasser, & Snell, 2016). The early‐successional ecosystem after a stand‐replacing disturbance is expected to show increased taxonomic diversity, as well as increased diversity of functional traits (Grime, 2006), as survivors, opportunists, and specialists exploiting new niches co‐exist (Swanson et al., 2011). Shade‐tolerant forest species often persist, with diversity increased by the addition of nitrophilous and light‐demanding pioneer species (Donato, Campbell, & Franklin, 2012; Hedwall & Brunet, 2016; Ilisson, Metslaid, Vodde, Jõgiste, & Kurm, 2006; Nováková & Edwards‐Jonášová, 2015; Winter et al., 2015). However, a continuing shift in vegetation community toward a different composition which diverges from unaffected areas could indicate an emerging alternate state. In this study, we use the opportunity provided by the combined disturbances at the Aneboda monitoring site to look for evidence of such a regime shift. The expected path of such a shift at this site would be via the increasing dominance of alternative late‐successional tree species capable of forming a new canopy, particularly *F. sylvatica*.

Naturally regenerating spruce forest can directly recover the tree composition found before disturbance with spruce dominating as both pioneer and late‐successional species (Heurich, 2009; Nováková & Edwards‐Jonášová, 2015), even where initial regeneration is sparse and most spruce have died (Kupferschmid, Brang, Schönenberger, & Bugmann, 2006; Kupferschmid & Schönenberger, 2002). Consequently, at Aneboda, spruce would be expected to remain the dominant tree species during regeneration in a resilient forest. However, the stock of small trees present under the canopy before disturbance can be decisive in determining the postdisturbance canopy composition (Messier et al., 1999). If these can survive the disturbance, they have an obvious advantage over seedlings once released from light limitation, provided they are of a species that can make use of these conditions (e.g., *Fagus*).

In the study area, the spatially heterogenous impact of the combined disturbances has resulted in a clear division of the plots at the site into affected and apparently unaffected areas. In affected areas, the damage is extreme, resulting in an effectively binary distinction between impacted plots and apparent refuges (unimpacted control plots). Refuges are defined as plots which maintained a mean percentage canopy cover of *P. abies* that was above the whole site mean value at all stages of the period since the disturbances began (see Methods). We hypothesize that the vegetation will develop into different vegetation communities in the refuges and the other plots, indicating a possible regime shift induced by the disturbances in the impacted areas.

This study was prompted by the rare opportunity provided by a combination of disturbance events (windthrow and beetle outbreak) affecting a site covered by an ongoing long‐term program of monitoring (ICP IM, 2018). The study aims to use inventories of the vegetation to investigate the following hypotheses that explore the resilience of boreal forest ecosystems:

1. That vascular plant species abundances, taxonomic and functional diversity, and community composition have significantly changed in the postdisturbance period,
2. That these changes show spatial and/or temporal patterns. Specifically, we hypothesize that refuge plots and nonrefuge plots will show differences in the variables investigated in hypothesis 1. We also hypothesize that successional change in community composition in the affected areas has occurred over time, and finally we aim to answer the question: Do changes found in the ground layer show evidence of ecosystem recovery or a postdisturbance regime shift?

## 2 Methods

### 2.1 Site description

**Figure 2:**
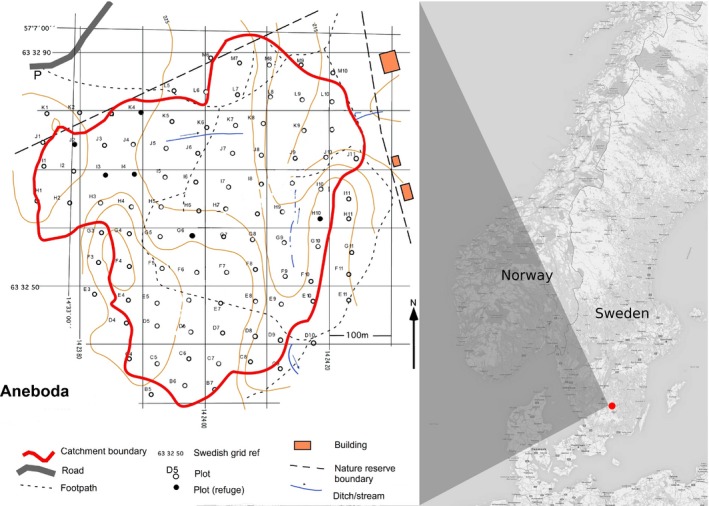
Location and layout of the Aneboda monitoring site

The study site, Aneboda, is located in the boreo‐nemoral zone in southern Sweden (N57°06´43”, E14°33´04”, Figure 2). The site is a 19‐ha catchment and has been woodland for several hundred years (Länsstyrelsen i Kronobergs län, 1998). The site has a long‐term average temperature of +5.8°C, average precipitation of 712 mm per year, an average snow cover of 110 days, and a vegetation period of 195 days. The dominant soil type is podzol, with a granite bedrock. Air pollutant deposition is around 8 kg ha^‐1^ year^‐1^ N and 3 kg ha^‐1^ year^‐1^ S (Löfgren et al., 2011). Hydrogeochemical research on the site began in the 1980s, and vegetation and soil assessments began in 1982. These activities were reorganized and the site became part of the ICP IM network from 1995, collecting a range of chemical and biological data (see (ICP IM (2018) for subprograms and protocols).

The site is thought to have been clear‐cut sometime in the 1860s, after which the present forest established spontaneously (Länsstyrelsen i Kronobergs län, 1998). There are very few signs of management, the area has been a protected area since 1997 and is also a NATURA 2000 site. The predisturbance forest (and in “refuge” areas at present) was dominated by Norway spruce (*P. abies*) with some broadleaved trees such as birch (*Betula*spp.) and beech (*F. sylvatica*), particularly in the shrub layer. The ground vegetation was dominated by *Vaccinium myrtillus* and carpets of mosses (mainly. *Dicranum* spp. and *Hylocomium splendens*) (Grandin, 2004).

Storm Gudrun felled around 20% of the trees in January 2005, and a subsequent bark beetle attack between 2008 and 2011 eliminated most mature spruce trees. By 2011, more than 50% of the trees with a diameter at breast height (DBH) of at least 25 cm were dead (Löfgren et al., 2014), and the die‐off of trees caused by the bark beetle has continued since then (J. Weldon, personal observation).

### 2.2 Vegetation monitoring

Vegetation monitoring is undertaken according to the protocols set out in the ICP IM manual (Manual for Integrated Monitoring, 1998), and the most relevant details are as follows. Every fifth year, the vegetation is surveyed in permanent circular 100‐m^2^plots arranged in a 50‐ by 50‐m grid (Figure 2) covering the whole catchment (Löfgren et al., 2011). In each plot, the percentage cover of all plant species present is recorded separately at each layer by visual estimates (from 1% to 100% cover). Layers are defined as follows: The tree layer is >5 m height, shrub layer is vegetation from 1 to 5 m height, and the ground layer is vascular plants under 1 m height. Total overall cover at each vegetation layer (tree, shrub, and ground, considered separately) is also recorded. At adjacent (to avoid trampling damage) circular 314‐m^2^ plots, the species, position, and diameter of all trees with a DBH ≥ 5cm were recorded, and for smaller trees (DBH < 5cm), the total number of individuals of each species was recorded. Vegetation data collected using the current protocol are available for the years 2006, 2011, and 2016 (data collected during the summer in all cases). The taxonomy follows Euro+Med PlantBase (2006).

The monitoring program was severely disrupted by the 2005 storm and subsequent bark beetle outbreak. Although 44 plots were accessible in 2006, increasingly difficult and dangerous access due to the accumulation of fallen trees in the following years meant that only 23 plots were continuously recorded throughout the postdisturbance period. These 23 plots are the focus of this study. Six of these 23 plots were identified as potential refuges (unimpacted control plots) meaning that *P. abies* maintained a mean percentage canopy cover that was above the whole site mean value (23% in 2006) at all stages of the postdisturbance period. The status of these plots as potential refuges was confirmed during a site visit in September 2017 (J. Weldon personal observation).

### 2.3 Data analysis

To explore changes in vegetation community composition over time and by refuge status, we applied nonmetric multidimensional scaling (nMDS), using the R package vegan 2.5‐1 (Oksanen et al., 2018) The nMDS analysis was applied to a Bray–Curtis dissimilarity matrix in all cases (Faith, Minchin, & Belbin, 1987). In all nMDS ordinations, a three‐dimensional space was selected and a minimum stress value of 0.2 was required.

We tested for differences in community composition over time, and between refuges and other plots, by using year and refuge status as factors in permutational multivariate analysis of variance (PERMANOVA (Anderson, 2001)) with the adonis2 function of the R package vegan 2.5‐1 (Oksanen et al., 2018). The BETADISPR function of vegan was used to test for homogeneity of multivariate dispersion, an assumption of PERMANOVA (although in balanced designs such as this study, PERMANOVA is robust to heterogeneity (Anderson & Walsh, 2013)).

To test for changes in functional diversity that could reflect changes in community composition, we used trait data acquired from the Biolflor (Kuhn & Klötz, 2002) and Ecoflora (Fitter & Peat, 1994) databases using the TR8 0.9.18 R package (Bocci, 2015).

Functional classifications used were Raunkiær life form (Raunkiaer, 1934) and classification in Grime's CSR model (Grime, 1977). The former is a relatively simple morphological characteristic, and the latter is based on plant strategies for dealing with stress and/or disturbance. Life form is related to response to disturbance (Cornelissen et al., 2003) while community‐weighted mean CSR strategy would be expected to reflect the changed abiotic conditions postdisturbance. Both are therefore relevant to investigating postdisturbance succession. To investigate possible changes in a range of environmental variables and in the range of exploited niches, per‐plot community‐weighted means of these values were calculated using the R package vegdata 0.9.1 (Jansen & Dengler, 2010). The FD 1.0‐12 R package (Laliberté & Legendre, 2010) was used to calculate community‐weighted means for several functional diversity indices: functional evenness (FEve), functional richness (FRic) (Villéger, Mason, & Mouillot, 2008), functional dispersion (FDis) (Laliberté & Legendre, 2010), and Rao's quadratic entropy (Q) (Botta‐Dukát, 2005). These indices provide different approaches to quantifying and summarizing the relationships between species in multidimensional functional trait space, that is, measuring the spread of points (species) in an n‐dimensional trait space. FDis and RaoQ estimate the dispersion of species, weighted by relative abundances, FRic is the volume occupied by the community, and FEve is the regularity of abundance distribution in this volume. Functional dispersion (FDis) and RaoQ are somewhat similar, and high positive correlations between the two are expected (Laliberté & Legendre, 2010). These results were compared across years and between refuges/other plots using ANOVA/Tukey post hoc testing following Levene's test for homogeneity of variances across groups.

A similar methodology was applied to analysis of community‐weighted mean Ellenberg values, in order to investigate community responses to postdisturbance changes in abiotic variables (light, pH, nutrient levels, and moisture). We acquired Ellenberg indicator values (Ellenberg, 1950) from the same databases as the functional trait data and compared community‐weighted mean values calculated with the FD package across refuge status and years. The use of Ellenberg values as a response variable is common, but has also been criticized (e.g., Zelený and Schaffers (2012)) and the appropriate statistical treatment is still debated. Here, we adopt the modified ANOVA permutation test of Zelený and Schaffers (2012), which is intended to avoid the tendency the authors note for biased results when Ellenberg values are related to species composition by accounting for compositional similarity inherited in mean Ellenberg values.

To examine which species best characterized communities and whether this changed with time and refuge status, we analyzed indicator species using the indval function of the R package labdsv 1.8 (Roberts, 2007). This is an adaptation of the method developed by Dufrêne and Legendre (1997), and calculates the indicator value of a given species as the product of its relative frequency and relative average abundance in clusters.

Changes in the abundances of individual species between the start and end of the study period, that is, between surveys performed in 2006 and 2016, were examined using two tailed *t* tests. As the same 23 plots were sampled on each occasion, these tests were paired.

All data analyses were done in R version 3.4.4 (R Core Team, 2018).

## 3 RESULTS

### 3.1 Changes in overall cover by layer

**Figure 3:**
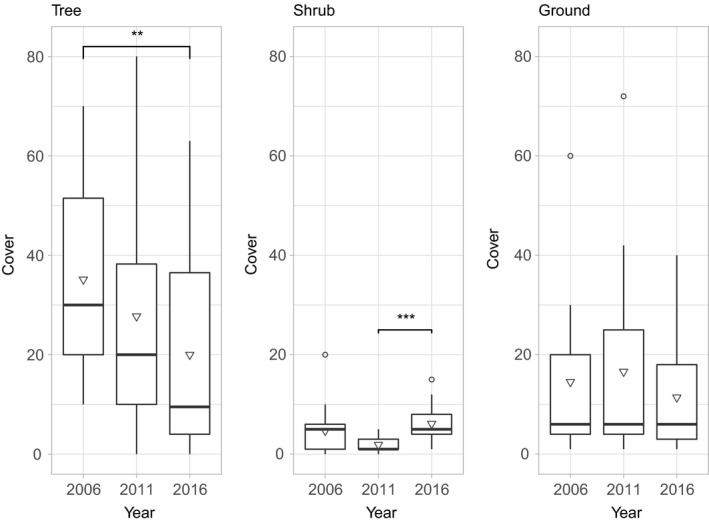
Between‐year changes in mean cover by layer (across all sampled plots).Upper and lower limits of the box are 75th and 25th percentile, respectively, horizontal bars represents the median, and triangles show mean values. Whiskers extend up to 1.5 times the interquartile range. Outliers beyond that distance shown by open circles. Bars and asterisks indicate significance differences (*p < 0.05, **p < 0.01, ***p < 0.001)

The mean tree layer cover of plots declined significantly (ANOVA, Tukey, *p* = 0.016) between 2006 and 2016, while the shrub layer saw a significant increase (ANOVA, Tukey, *p* = 0.005) in cover from 2011 to 2016. The mean ground layer cover of plots showed no significant changes (Figure 3).

### 3.2 Within layer changes

In the ground layer, there was a significant difference in community composition between refuge and nonrefuge plots. However, differences among years were restricted to plots affected by the disturbances. In the shrub layer, the only significant result found was in community composition between refuge and nonrefuge plots. In the tree layer, significant differences were found in both community composition and multivariate dispersion between both refuge and nonrefuge plots, and between years for all plots taken together. Nonrefuges showed a significant change in community composition between years while refuges did not (Table 1).

**Table 1:** PERMANOVA and Betadisper test results for differences in community composition and multivariate dispersion, with year and refuge status as factors. Tests were performed on all plots together, and separately on refuges/nonrefuges only

	Permanova		Betadisper	
	Refuge	Year	Refuge	Year
Ground layer				
All plots	***	NS	***	NS
Refuges	na	NS	na	NS
Nonrefuges	na	*	na	NS
Shrub layer				
All plots	*	NS	NS	NS
Refuges	na	NS	na	NS
Nonrefuges	na	NS	na	NS
Tree layer				
All plots	**	*	***	*
Refuges	na	NS	na	NS
Nonrefuges	na	***	na	NS

Note

Asterisks indicate a significant result. “NS” indicates a nonsignificant result, “na” indicates test not performed for this combination of plots and factor.

*p < 0.05; **p < 0.01; ***p < 0.001.

#### 3.2.1 Ground layer

**Figure 4:**
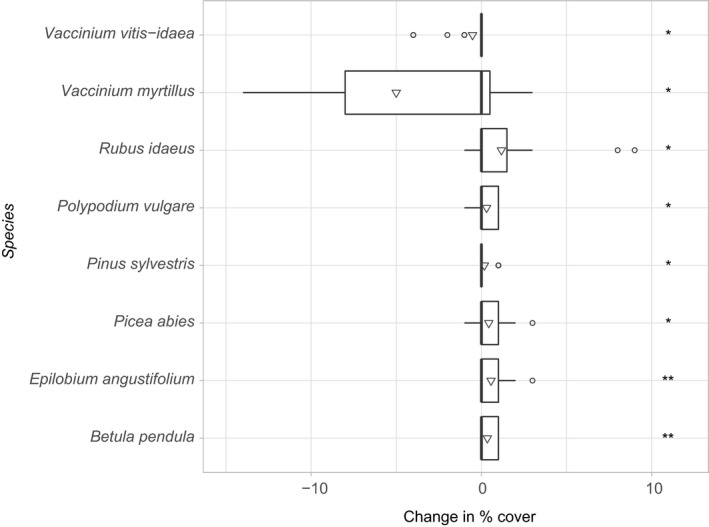
Significant changes in percentage cover of vascular plant species in the ground layer 2006–2016. Upper and lower limits of boxes are 75th and 25th percentile, respectively, vertical bars represent the median, and triangles show mean values. Whiskers extend up to 1.5 times the interquartile range. Outliers beyond that distance shown by open circles. Asterisks indicate significance differences (*p < 0.05, **p < 0.01)

In many cases, changes in individual species abundances between 2006 and 2011 are partially or completely reversed from 2011 to 2016, or occur almost entirely in one period, with few species showing consistent increase or decrease across both periods (Supporting Information Figure S1, Appendix S1). Nevertheless, there were significant changes (paired *t* tests) in the abundance of eight species between 2006 and 2016 (Figure 4). (Note that according to the sampling protocol, species cover <1% is noted as 1% (=1 m^2^). However, in many cases, the true cover is considerably less. Some species constitute only 0.01% (=10 × 10 cm) cover or less (pers. obs. by field staff). In the ground vegetation data, 1% is the most frequent cover. Consequently, percentage changes in cover between surveys appear very small but are likely underestimates for many of those species with an initial noted cover of 1%.

#### 3.2.2 Shrub layer

There were no significant changes within the shrub layer between 2006 and 2016 when comparing individual species abundances. However, this obscures a change in *P. abies* cover. Between 2006 and 2011, there was a significant decrease (paired *t* test *p* = 0.009) followed by a significant increase (paired *t* test, *p* = 0.0001) between 2011 and 2016. The overall net effect of no significant change for *P. abies*is therefore a result of cover being reduced and then bouncing back following the disturbances.

While year was not a significant factor in the shrub layer, there was a significant difference in community composition by refuge status (*p* = 0.03, PERMANOVA, Table 1). While mean cover of *P. abies* in both refuges and nonrefuges was at a similar level (3.47% in refuges and 3.07% for nonrefuges), the cover of many deciduous species was higher in nonrefuges, notably that of *Betula pubescens,* *Betula pendula,* and *F. sylvatica*, although these did not emerge as significant in the paired *t* tests.

#### 3.2.3 Tree layer

Both *P. abies* and *Pinus sylvestris* showed a significant decline in cover between 2006 and 2016 (paired *t* test, *p* = 0.008 and *p* = 0.02, respectively). *P. abies* declined from a mean cover of 23.5% in 2006 to 14.6% in 2016.

There were significant differences in community composition both between years (*p* = 0.008) and between refuge plots and nonrefuges (*p* = 0.001) while nonrefuges (but not refuges) were significantly different in their community composition between years (*p* = 0.001) (PERMANOVA analysis, Table 1).

There was a significant difference in community composition between refuges and nonrefuges when taking all plots together (PERMANOVA, *p* = 0.001) while difference between years was not significant (*p* = 0.08) (Table 1). However, taking refuges and nonrefuges separately with year as a factor showed a significant difference between years for nonrefuges (PERMONOVA, *p* = 0.04), but not for refuges (*p* = 0.93) (Table 1).

#### 3.2.4 Ground layer

**Figure 5:**
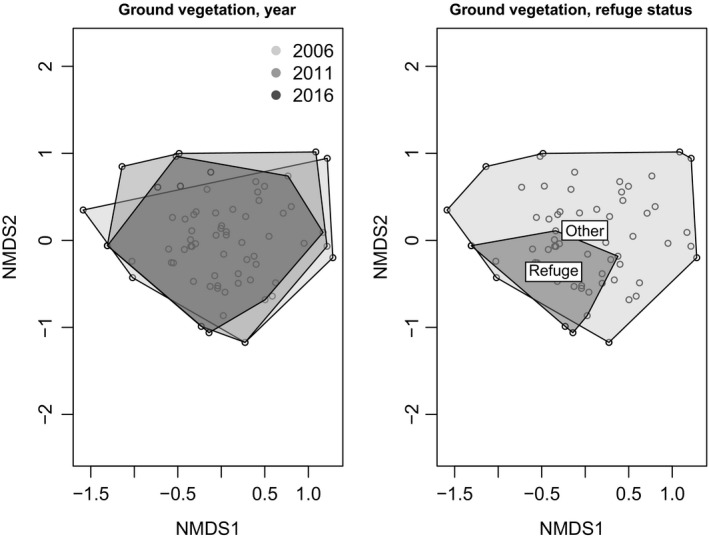
nMDS of ground layer vegetation plots showing convex hulls for survey years (left) and refuge status (right) shows considerable overlap. Convex hulls drawn from points representing plots, based on Bray–Curtis dissimilarity, stress 0.17

An nMDS of the ground layer vegetation showed no clear separation between years, while a grouping according to refuge status shows an almost complete overlap of the refuge and nonrefuge plots (Figure 5).

**Figure 6:**
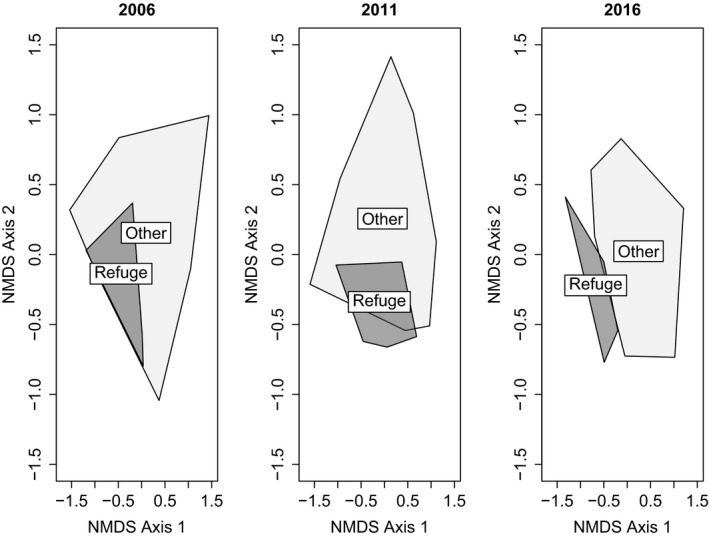
nMDS of ground layer plots with convex hulls indicating refuges and nonrefuges, showing an increasing separation of refuges and nonrefuges over time, convex hulls drawn from points representing plots, Bray–Curtis dissimilarity (stress 0.11, 0.12, 0.11)

However, an increasing separation between refuges and nonrefuges is revealed with year‐by‐year NMDS analysis using refuge status as a factor, with a clear distinction having emerged by 2016 (Figure 6).

### 3.3 Indicator species

Indicator species analysis was undertaken on the ground layer data to find which species best characterized the different factor groupings (Table 2). *V. myrtillus* was the only significant indicator for the community in 2006. The community in 2011 did not have any significant indicators, and the 2016 community indicators were three ruderal taxa and *P. abies* indicating a recovery of the spruce. The refuge plots had only one significant indicator species, while the nonrefuge plots had five significant indicators but most with relatively low indicator values around 0.3 (Table 2).

**Table 2:** Significant ground layer indicator species for different years and refuge status

Species	Group	indval	*p*	Frequency
*Vaccinium myrtillus*	2006	0.54	0.013	66
*Picea abies*	2016	0.41	0.021	52
*Rubus idaeus*	2016	0.31	0.031	22
*Epilobium angustifolium*	2016	0.30	0.008	13
*Epliobium spp*.	2016	0.17	0.029	4
*Dryopteris carthustiana*	Not refuge	0.41	0.049	31
*Betula pubescens*	Not refuge	0.38	0.032	28
*Oxalis acetosella*	Not refuge	0.33	0.018	17
*Betula pendula*	Not refuge	0.29	0.011	15
*Epilobium angustifolium*	Not refuge	0.26	0.03	13
*Maianthemum bifolium*	Refuge	0.41	0.005	20

### 3.4 Biotic‐abiotic associations

**Figure 7:**
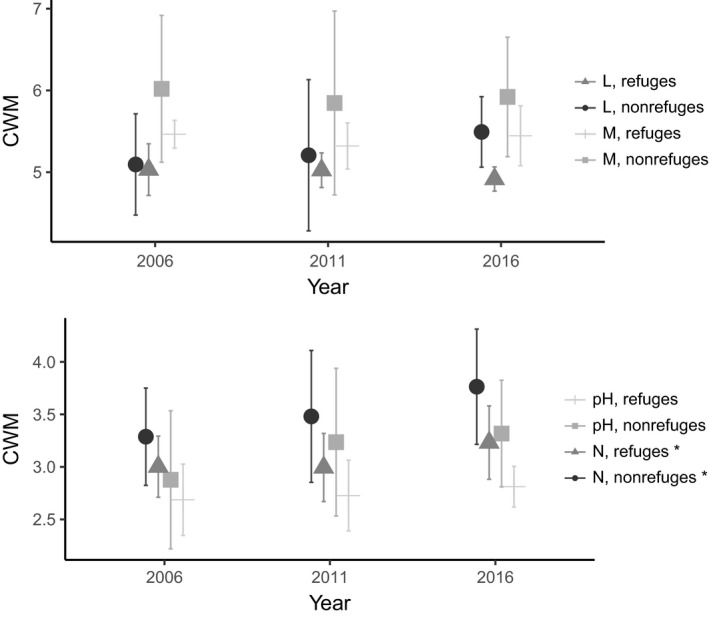
Community‐weighted mean (CWM) Ellenberg values (L = light, M = moisture, N = nutrients, pH = pH), changes over time with refuges and nonrefuges plotted separately. Points for the same year have been spaced to avoid overlapping error bars (*SD*). N refuges significantly higher overall than N nonrefuges (*p < 0.05)

Light (L), moisture (M), pH, and nitrogen (N) mean Ellenberg values are all lower in the refuges, and an increasing divergence in mean L value can be seen between refuges and other plots (Figure 7). While no significant differences were found between years (permutational ANOVA (Zelený and Schaffers (2012)), taking data from all years together nonrefuges had a significantly higher *N* value than refuges (*p* = 0.04).

### 3.5 Functional diversity

Mean community‐weighted values for classifications in two functional groupings (life form and CSR strategy) associated with response to disturbance were calculated and tested for difference between years. There were significant increases in functional dispersion (*p* = 0.01), and Rao's Q (*p* = 0.03), but no significant change in functional evenness or functional richness. These changes were driven by the nonrefuge plots, as no significant changes were found within refuges (Table 3).

**Table 3:** Changes in functional diversity indices (functional dispersion (FDis), evenness (FEve), and Rao's quadratic entropy [RaoQ]). Tested using ANOVA/Kruskal–Wallis with year as grouping)

	FEve	FDis	FRic	RaoQ
All plots	ns	*	ns	*
Refuges	ns	ns	ns	ns
Nonrefuges	ns	**	ns	**

*p < 0.05; **p < 0.01

### 3.6 Taxonomic diversity

There was a significant increase in taxonomic diversity with time, a change driven by the nonrefuge plots (Table 4). Mean species richness across all plots also rose from 8 in 2006 to 10.7 in 2016, and again the nonrefuges contributed to this rise with the mean increasing from 8.3 in 2006 to 12.2 in 2016, while refuges showed no increase.

**Table 4:** Mean Shannon diversity index values by year and refuge status, standard deviations in brackets

	2006	2011	2016	Difference (ANOVA)
All plots	1.61 (0.47)	1.89 (0.44)	2.06 (0.46)	**
Refuges	1.78 (0.36)	1.78 (0.46)	1.61 (0.56)	NS
Nonrefuges	1.55 (0.50)	1.94 (0.44)	2.21 (0.30)	***

**p < 0.01; ***p < 0.001.

### 3.7 Small trees

No significant differences in community composition were found between refuge and nonrefuge plots or between years when analyzing only the small tree community, that is, woody vegetation with a DBH of < 5cm (PERMANOVA) (Table 5). Nor were any significant changes found in the abundances of individual species between years (paired *t* tests), likely due to the extreme heterogeneity of abundances between plots (e.g., mean coefficient of variation for *F. sylvatica* is 219%), but the results show an almost tenfold increase in count of small *F. sylvatica. P. abies* however remains by far the most abundant species across the whole postdisturbance period (Table 5) and increases in abundance between 2011 and 2016 after a decrease between 2006 and 2011.

**Table 5:** Mean number of trees <5 cm diameter counted per plot, standard deviations in brackets

	2006	2011	2016
*Picea abies*	19.4 (12.64)	14.0 (10.29)	15.4 (11.71)
*Fagus Sylvatica*	0.15 (0.38)	0.54 (1.13)	1.46 (2.85)
*Betula pendula*	0.38 (0.96)	0.08 (0.28)	2.08 (4.79)
*Betula pubescens*	1.38 (2.29)	0.08 (0.28)	2 (3.39)
*Sorbus aucuparia*	0.62 (1.33)	1.08 (2.63)	1.77 (4.19)
*All deciduous*	4.15 (3.89)	4.85 (5.91)	9.46 (15.66)

Note

Some species with very low abundances omitted.

## 4 DISCUSSION

Overall community composition has changed postdisturbance, with increases in ruderal species, in deciduous tree species, in taxonomic and functional diversity, and in mean Ellenberg N values (i.e., plant–environment associations shaped by nutrient levels), as suggested in our first hypothesis. In agreement with our second hypothesis, these changes are mostly only present in the nonrefuge plots, while nonrefuge plots also show change in community composition over time. However, even in disturbed areas, *P. abies* appears to be recovering strongly, suggesting ecosystem recovery rather than a postdisturbance regime shift.

There is clear evidence of changes in community composition since the disturbances. While the nMDS ordinations of ground layer vegetation show no clear change over time across all plots taken together (Figure 5), PERMANOVA analysis shows (Table 1) that year is a significant factor in both the ground and tree layers. Ground layer vegetation functional diversity showed an increase across the site in functional dispersion and Rao's Q (Table 3). Given that the functional groupings chosen for analysis are associated with response to disturbance, this is likely a result of the increase in disturbance adapted species making use of the niches created by the perturbations, while the previous forest floor species continue to persist in the ground layer. The increase in disturbance adapted species alongside the continued presence of forest species typical of later successional stages was also expected to result in increased taxonomic diversity (Ilisson et al., 2006; Swanson et al., 2011; Uotila & Kouki, 2005) which is indeed shown by the comparison of mean Shannon values in the ground layer (Table 4).

The observed changes in the vegetation community are related to time since the disturbances began but also to the status of plots as refuges/nonrefuges. The ground layer vegetation shows a clear distinction between refuge plots and nonrefuges, with only the latter showing significant changes in community composition with time. Both the changes in individual species abundances and indicator species suggest that this shift in community composition is driven by declines in the species typical of the predisturbance forest floor, that is, the dwarf shrubs *Vaccinium myrtillus* and *Vaccinium vitis‐idaea*. In tandem, the abundances of species associated with colonizing the open spaces created by disturbance have increased (for example *Rubus idaeus, Epilobium angustifolium, Betula*spp.). However, *P. abies* has also increased in abundance. Given that refuges were defined by maintaining a high level of spruce in the canopy, it was expected that *P. abies* seedlings would have a relatively high abundance in refuges, but they are in fact widespread across the site.

The nonrefuge communities showed a higher value for their mean preference for N than those in the refuges. This response is unsurprising, as large quantities of N are made available by a disturbance such as this. Litter increases as trees die, demand from trees for available nitrogen is simultaneously reduced, and N deposition previously directly taken up by mature spruce is available for ground‐level vegetation (Karlsson, Akselsson, Hellsten, & Karlsson, 2018). This increased available N pool is made use of by ruderal herbaceous and shrub species (which additionally benefit from the change in light regime) but can also result in increased leaching (Karlsson et al., 2018). At Aneboda, the amount of N taken up postdisturbance by the previously N limited vegetation community has meant that the leakage of N from the site has been very limited compared to similarly disturbed N saturated sites elsewhere (Löfgren et al., 2014; Mikkelson et al., 2013).

A significant increase in the functional diversity indices has occurred only in the nonrefuge plots (Table 3), and the increase in taxonomic diversity (Table 4) is also only seen in the nonrefuges, again suggesting that the sites hypothesized to be refuges have been resistant to the changes affecting the nonrefuges.

The nMDS results demonstrate an increasing separation between plots identified as refuges and the nonrefuge plots (Figure 6). In conjunction with other results outlined above, this shows that the hypothesized refuges are indeed functioning as such, with a substantially preserved predisturbance vegetation community despite their obvious susceptibility to edge effects in this heterogeneously disturbed habitat. This surprising persistence can be conceived of as a form of conservative ecological memory of the previous ecosystem state enhancing the ecological resilience of the forest (Allen et al., 2016; Jõgiste et al., 2017; Johnstone et al., 2016). At the same time, the nonrefuges have moved in a direction which is more typical of postdisturbance community composition.

While the results outlined above are clear regarding the differences over time and between refuges/nonrefuges, the question of whether these changes are evidence of a regime shift or not is more nuanced. The impact of the disturbances at the Aneboda monitoring site is most immediately obvious in the tree layer, with a large decline in overall cover, driven by a reduction in the abundance of *P. abies* outside the refuges (Figure 3). This gap creation presents opportunities for species able to take advantage, such as the shade‐tolerant seedlings/saplings able to grow under the previous canopy. While fire eliminates this potential canopy in waiting, bark beetle and storm perturbations do not (Kupferschmid & Schönenberger, 2002). Although tall shrub cover is generally sparse in Scandinavian spruce forest (Boonstra et al., 2016), the individuals present in this layer can be released from light limitation by disturbance and grow rapidly (Kupferschmid & Schönenberger, 2002; Messier et al., 1999). The potential opportunity for *Fagus*at the study site is clear, but is the site moving to a new, deciduous‐dominated state?

The differences demonstrated between refuges and nonrefuges, and particularly the increasing separation in the ground layer of the two types of plots over time, are compatible with the hypothesis that the disturbed areas are developing a different vegetation community, dominated by deciduous tree species. The changed conditions in the disturbed areas have clearly provided opportunities to species able to take advantage (of, for example, increased nutrients and light levels), resulting in shifts in community composition. Deciduous tree species have increased in abundance (Table 5). However, the unexpectedly widespread distribution and high cover of *P. abies* in the disturbed areas show that spruce is successfully recolonizing there from less disturbed areas. *P. abies* does not persist long in the seedbank (Rydgren & Hestmark, 1997), and the high levels of ground layer spruce seedlings in the disturbed areas must have originated from unaffected areas, at least in the later surveys.

In the shrub layer results, we see a significant decrease and subsequent recovery of *P. abies,*which as the dominant species is also reflected in the changes in overall shrub layer cover (Figure 3). The nonrefuge sites differ from the refuges by having a higher cover of deciduous species rather than significantly less *P. abies*, postdisturbance. Spruce has maintained its presence across the site in the shrub (and ground) layer. Analysis of the distribution of small trees (stem diameter < 5cm) is another way to consider which species were available to benefit from disturbance. *Ips typographus*requires host trees with a bark thickness of at least 2.5 mm and strongly prefers mature trees (Grunwald, 1986) so we would expect to find small *P. abies* individuals of this size class surviving even in areas affected by bark beetle infestations. While there is a clear increase in the numbers of *F. sylvatica, Betula*spp., and* Sorbus aucuparia* found, *P. abies* remains the most abundant species among small trees by an order of magnitude in all years (Table 5).

An increase in pioneer tree species typical of postdisturbance succession in boreo‐nemoral spruce forest, such as *Betula*spp., is unlikely to point to a regime shift. Even in situations where they dominate the initial canopy postdisturbance, shade‐tolerant spruce will eventually outcompete them. An increasing dominance of deciduous species capable of forming an alternative late‐successional canopy (e.g., *F. sylvatica*) could however indicate an impending regime shift, but despite an increase in numbers and cover of *F. sylvatica,*it does not seem to be outcompeting *P. abies*. On the contrary, while *P. abies* was the species most adversely affected by disturbance at a canopy level, it has appeared abundantly postdisturbance in the shrub and ground layers, and in the small tree surveys, suggesting the spruce‐dominated forest will persist. Although *F. sylvatica* is a strong competitor for light with other canopy species (Ligot, Balandier, Fayolle, Lejeune, & Claessens, 2013) (the conclusion of which could take decades to become apparent), it is here near the northern limit of its range. While beech has been observed to displace spruce as the postdisturbance dominant species in this region, it seems to require a strong understory presence awaiting release (Bolte et al., 2014), which our results suggest was insufficient at Aneboda. Given the results found, we would expect the observed divergence between refuges and nonrefuges in the ground layer to reverse as the relatively abundant spruce grow and ground layer conditions under them gradually become more similar to the predisturbance regime. However, this can be a slow process. A decline in cover and richness of early‐successional species in a spruce forest in Finland, for example, was seen only 20 years after disturbance (Merilä, & Jortikka, 2013).

We can identify several factors likely to have contributed to this apparently strong recovery. While shade‐tolerant *P. abies* is better able than light‐demanding species to recolonize small gaps in forests similar to this (Liu & Hytteborn, 1991), larger areas can be challenging. Dispersal rates and the size of the disturbed area are key in recovery after perturbation (van de Leemput, Dakos, Scheffer, & Nes, 2018), and seed dispersal is strongly linked to proximity to surviving forest edge (Rozman, Diaci, Krese, Fidej, & Rozenbergar, 2015). It seems likely that the survival of areas able to function as refuges and the patchy nature of the disturbance impact have been essential in allowing rapid recolonization at Aneboda by the previously dominant tree species*, P. abies*. The growth of spruce seedlings is also strongly facilitated by dead wood (Gratzer & Waagepetersen, 2018), while postdisturbance clearance of this dead organic matter can result in the emergence of a birch‐dominated pioneer woodland instead (Fischer, Lindner, Abs, & Lasch, 2002). Spruce seedlings are shallow‐rooted and relatively slow‐growing, making them poor competitors against ground vegetation postdisturbance unless there is coarse woody debris available to provide a seedbed (Jonášová & Prach, 2004; Rozman et al., 2015). The hands‐off management strategy at Aneboda has resulted in a high abundance of dead wood postdisturbance which has also likely contributed to the observed recovery. Another possible factor affecting recovery is that wind damage and insect attack are in some respects redundant disturbances. The immediate impact of both is on the canopy, while the understorey and soil are much less directly affected. The conceptual model of Roberts (2004) suggests that combined disturbances that “overlap” in this way are less challenging to forest resilience than those which complement one another (e.g., wind and fire can together simultaneously affect all three layers, creating a much more difficult environment for recovery, and a greater probability of an alternate state emerging).

To more explicitly frame the results in a resilience theory framework, we can say that the system has remained within one basin of attraction (i.e., has not undergone a regime shift). Such a recovery is in itself evidence of only “engineering” resilience, that is, a return to predisturbance conditions in a system with a single equilibrium (Angeler & Allen, 2016). However, a shift to a beech‐dominated state was a real possibility (i.e., there is probably more than one basin of attraction in this system). Given this multiple basin of attraction context, we can interpret the observed recovery as evidence of ecological resilience in the system.

Our results also show the importance of monitoring programs over the medium and long term. While initial regeneration after disturbance can be used to predict later successional pathways, combined disturbances can complicate this predictive property. A North American study found initial regeneration after wind damage strongly predictive of vegetation community 10 years later, but a combined disturbance (wind and fire) resulted in initial regeneration with very poor predictive properties (Gill, Jarvis, Veblen, Pickett, & Kulakowski, 2017). In the current study, changes in the relative abundances of many common species between 2006 and 2011 suggested a consistent trend in community composition. However, with the benefit of data from the 2016 survey, we can see that in many cases these changes leveled out or reverted toward their predisturbance means (Supporting Information Figure S1, Appendix S1). This demonstrates both the potential problems with conclusions based on changes observed over relatively short time periods and the value of the long‐term data sets provided by monitoring programs in avoiding them. While the data used here are perhaps best characterized as medium term, the value of the ICP IM and similar monitoring programs will only increase as they continue into the future.

## AUTHORS' CONTRIBUTIONS

UG conceived the ideas; JW and UG designed methodology; JW and UG analyzed the data; and JW led the writing of the manuscript. Both authors contributed critically to the drafts and gave final approval for publication.

## Data Availability

Data used in this study are freely available at http://info1.ma.slu.se/IM/data.html.
