# Supplementary material for: Major disturbances test resilience at a long‐term boreal forest monitoring site
Source: Ecol Evol. 2019 Mar 15;9(7):4275–88. doi: 10.1002/ece3.5061 (PMC6467845; doi:10.1002/ece3.5061)
Supplement: Supplementary file 1 [file ECE3-9-4275-s001.docx]

**Supporting Information for review and publication**

**Appendix**


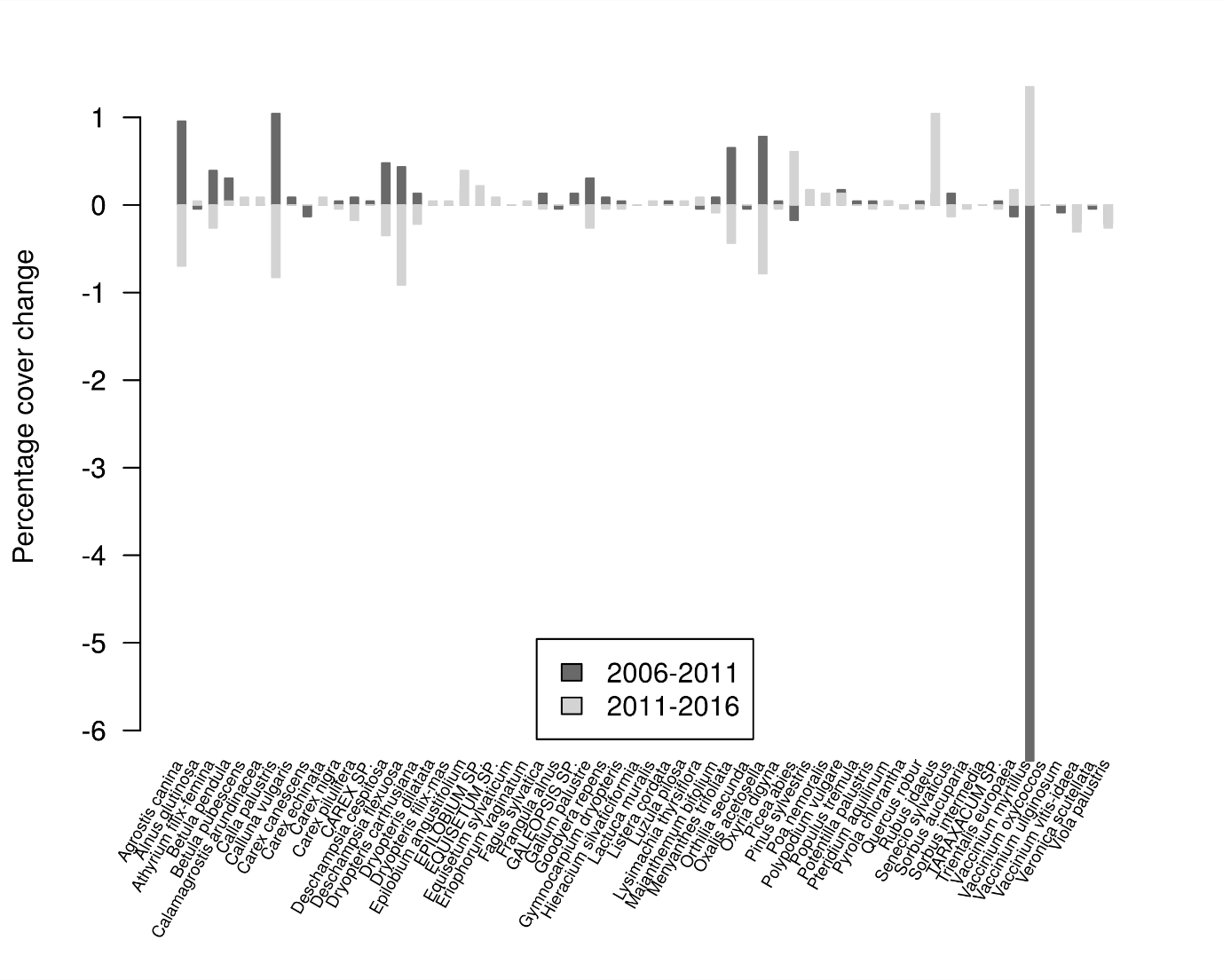


Figure 1: Changes in mean ground layer cover by species, compared between 2006 and 2011 as well as between 2011 and 2016.

Table 1: Constancy table for ground layer vegetation (only species found in at least 5 plots listed)

|  | 2006 | 2011 | 2016 |
| --- | --- | --- | --- |
| Agrostis canina | . | 0.22 | 0.17 |
| Athyrium filix-femina | . | 0.13 | 0.17 |
| Betula pendula | . | 0.30 | 0.35 |
| Betula pubescens | 0.39 | 0.39 | 0.43 |
| Calluna vulgaris | 0.22 | 0.26 | 0.22 |
| Carex canescens | 0.13 | . | . |
| Carex echinata | . | . | 0.13 |
| Deschampsia flexuosa | 0.70 | 0.70 | 0.74 |
| Dryopteris carthusiana | 0.39 | 0.43 | 0.52 |
| Epilobium angustifolium | . | 0.17 | 0.39 |
| Epilobium Sp. | . | . | 0.17 |
| Fagus sylvatica | 0.39 | 0.43 | 0.48 |
| Galeopsis Sp. | . | 0.13 | 0.13 |
| Luzula pilosa | 0.43 | 0.39 | 0.48 |
| Maianthemum bifolium | 0.26 | 0.35 | 0.26 |
| Oxalis acetosella | 0.22 | 0.22 | 0.30 |
| Picea abies | 0.78 | 0.61 | 0.87 |
| Pinus sylvestris | . | . | 0.17 |
| Polypodium vulgare | . | 0.22 | 0.35 |
| Pteridium aquilinum | 0.22 | 0.22 | 0.22 |
| Quercus robur | . | 0.13 | . |
| Rubus idaeus | 0.22 | 0.30 | 0.43 |
| Senecio sylvaticus | . | 0.13 | . |
| Sorbus aucuparia | 0.43 | 0.43 | 0.39 |
| Trientalis europaea | 0.22 | . | 0.26 |
| Vaccinium myrtillus | 0.91 | 0.96 | 1.00 |
| Vaccinium vitis-idaea | 0.70 | 0.65 | 0.61 |
| Viola palustris | 0.13 | 0.13 | 0.13 |

Table 2: Constancy table for shrub layer vegetation (only species found in at least 5 plots listed)

|  | 2006 | 2011 | 2016 |
| --- | --- | --- | --- |
| Alnus glutinosa | 0.05 | 0.06 | 0.05 |
| Betula pendula | 0.05 | 0.18 | 0.15 |
| Betula pubescens | 0.21 | 0.18 | 0.20 |
| Betula Sp. | 0.00 | 0.06 | 0.05 |
| Fagus sylvatica | 0.16 | 0.18 | 0.20 |
| Frangula alnus | 0.05 | 0.06 | 0.05 |
| Juniperus communis | 0.05 | 0.06 | 0.00 |
| Picea abies | 0.84 | 0.88 | 0.95 |
| Populus tremula | 0.00 | 0.06 | 0.05 |
| Quercus robur | 0.00 | 0.00 | 0.05 |
| Salix aurita | 0.05 | 0.06 | 0.05 |
| Salix Sp. | 0.00 | 0.00 | 0.05 |
| Sorbus aucuparia | 0.16 | 0.12 | 0.10 |
